# Resilience, Stress, Stigma, and Barriers to Mental Healthcare in U.S. Air Force Nursing Personnel

**DOI:** 10.1097/NNR.0000000000000182

**Published:** 2016-09-01

**Authors:** Stephen H. A. Hernandez, Brenda J. Morgan, Mark B. Parshall

**Affiliations:** **Stephen H. A. Hernandez, Lt Col, USAFR, NC, PhD, RN,** is Assistant Professor, College of Nursing, University of New Mexico, Albuquerque.; **Brenda J. Morgan, Col, USAF, NC, PhD, RN,** is Director, 59th Medical Wing Nursing Research Division, Joint Base San Antonio–Lackland, Texas.; **Mark B. Parshall, PhD, RN, FAAN,** is Professor, College of Nursing, University of New Mexico, Albuquerque.

**Keywords:** mental health services, military personnel, nursing, psychological resilience, social stigma

## Abstract

**Background:**

Stigma may deter military service members from seeking mental health (MH) services. Previously, substantial proportions of U.S. Air Force (USAF) registered nurses and medical technicians reported concerns about stigma with accessing MH services; in particular, that unit members might lose confidence in them or perceive them as weak, unit leadership might treat them differently, or accessing care might affect career advancement.

**Objective:**

This study assessed the extent to which stigma and barriers to accessing MH services as perceived by USAF nursing personnel are associated with resilience, stress, previous deployment, or demographic characteristics.

**Methods:**

An anonymous, online survey was administered to active-duty USAF registered nurses and medical technicians at three locations (*N* = 250). The survey included demographic items, the Stigma and Barriers to Care scales, Conner–Davidson Resilience Scale, and Perceived Stress Questionnaire.

**Results:**

Mean resilience was high, and perceived stress was moderate. About half of participants agreed that *unit members might have less confidence in me* (54%) or *unit leadership might treat me differently* (58%). Many also had concerns that *it would harm my career* (47%), *I would be seen as weak* (47%), or *there would be difficulty getting time off work for treatment* (45%). Stigma was positively correlated with perceived stress (*r* = .40, *p* < .01) and negatively correlated with resilience (*r* = −.24, *p* < .01). Officers had significantly higher stigma and resilience scores and lower stress scores compared with enlisted personnel, but those differences were small.

**Discussion:**

This study validated previous findings that substantial percentages of USAF nursing personnel have concerns that accessing MH services may adversely affect their careers and how they are viewed by unit leaders and peers. In addition, higher levels of concern about stigma were associated with higher levels of stress and lower levels of resilience. Limitations included a low response rate (18%) and self-selection biases.

Stigma may deter military service members from seeking mental health (MH) services (Castro & McGurk, 2007; Gorman, Blow, Ames, & Reed, 2011; Hoerster et al., 2012; Kim, Britt, Klocko, Riviere, & Adler, 2011). To date, only one single-site study of U.S. Air Force (USAF) nursing personnel focused on assessing perceptions of stigma and barriers to accessing MH services (Hernandez, Bedrick, & Parshall, 2014). USAF nursing personnel include registered nurses (RNs) who are commissioned officers with a minimum of a bachelor’s degree in nursing and enlisted medical technicians who function under the direct supervision of RNs or other healthcare providers (*Air Force instruction 46-101,* 2015). In that study, more than 50% of respondents agreed that accessing MH services might cause others to have less confidence in them and unit leadership might treat them differently, and more than 40% were concerned that seeking MH care would harm their careers or that others would perceive them as weak (Hernandez et al., 2014). Approximately 40% of nursing personnel reported difficulties with getting time off from work for treatment (Hernandez et al., 2014).

To help service members cope with stress and prevent the development of MH disorders, the U.S. Department of Defense has implemented programs to increase service members’ resilience. In a sample of USAF medical personnel preparing to deploy to Iraq, a higher level of resilience was related to a positive military experience and was negatively correlated with predeployment stressors, symptoms of posttraumatic stress disorder (PTSD), and negative affectivity (Maguen et al., 2008). Study findings with Iraq and Afghanistan veterans support that a higher level of resilience was associated with decreased risk for MH concerns (Hourani et al., 2012). In two studies of veterans, participants with PTSD reported significantly lower resilience compared with the participants without PTSD (Green, Calhoun, Dennis, Mid-Atlantic Mental Illness Research, Education and Clinical Center Workgroup, & Beckham, 2010; Pietrzak, Russo, Ling, & Southwick, 2011). A lower level of resilience was also reported by veterans who screened positive for depression, alcohol abuse, and psychosocial difficulties and was associated with suicidal ideation (Pietrzak et al., 2010; Pietrzak & Southwick, 2011).

## Purpose

Despite the importance of USAF nursing personnel in providing care to service members, only one study has investigated USAF nursing personnel’s perceptions of stigma and barriers to accessing MH care, and no studies have assessed associations of stigma and barriers with stress or resilience. Therefore, the specific aim of this study was to assess stigma and barriers to accessing MH services, stress, and resilience among USAF RNs and medical technicians. To accomplish this aim, the following research questions were asked:

1. What are USAF nursing personnel’s levels of stigma and barriers to accessing MH services, stress, and resilience?
2. What are the magnitude and direction of associations among stigma and barriers to accessing MH services, stress, and resilience in USAF nursing personnel?
3. Are demographic characteristics, military grade, past deployment, and access to MH services related to stigma and barriers to accessing MH services, stress, and resilience among USAF nursing personnel?

## METHODS

### Participants and Procedure

A cross-sectional, online survey was administered to USAF RNs and medical technicians at three sites. Sample size estimates, using G*Power 3.1 (Faul, Erdfelder, Buchner, & Lang, 2009), used assumptions based on results of the preliminary study (Hernandez et al., 2014), in which small, but statistically significant, differences between RN and medical technicians were found, corresponding to a point-biserial *r* ≈ .15 to .20 for the Stigma and Barriers scales. A sample size of 278 was sufficient for 80% power to detect an effect of that magnitude with a Type I error rate of .05 (two-tailed).

The study received institutional review board approval as exempt research. The survey was administered via the encrypted Research Electronic Data Capture (REDCap) Web portal (Harris et al., 2009) at the principal investigator’s institution (National Institutes of Health Grant UL1TR001449). With concurrence from the USAF Surgeon General’s office and local Chief Nurses’ support, the principal investigator traveled to each site to inform USAF nursing staff about the study. Site liaisons then sent an initial contact and three reminder e-mails via local distribution lists. Each e-mail included study information and instructions for completing the anonymous electronic survey. A recipient could forward these e-mails to a personal account, if desired. After reading an institutional review board-approved consent letter for anonymous surveys, service members who agreed to participate by clicking on the Web link were redirected to the survey in REDCap.

### Measures

The survey comprised demographic items, questions on access to MH services and military grade; the Stigma Scale (Britt, 2000; Hoge et al., 2004); the Barriers to Care Scale (Hoge et al., 2004); the Connor–Davidson Resilience Scale (CD-RISC; Connor & Davidson, 2003); and the Perceived Stress Questionnaire (PSQ; Levenstein et al., 1993). All scales, except the PSQ, had been used with service members and had shown adequate reliability and validity in multiple populations, as cited below.

#### Stigma and Barriers to Care

Each item in the Stigma and Barriers to Care scales uses Likert-type response options ranging from 1 = *strongly disagree* to 5 = *strongly agree*. Scores are calculated by summing item scores and dividing the score by the number of responses (Hoge et al., 2004; Kim, Thomas, Wilk, Castro, & Hoge, 2010). Score reliability estimated using Cronbach’s *α* was .89 to .95 (Britt et al., 2011; Hernandez et al., 2014; Kim et al., 2010, 2011), and the Barriers to Care Scale’s reported Cronbach’s *α* was .66 to .90 (Britt et al., 2011; Hernandez et al., 2014; Kim et al., 2010).

#### Resilience

Each item in the CD-RISC uses a rating from 0 = *not true at all* to 4 = *true nearly all the time* (Connor & Davidson, 2003). Scores are the sum of all 25 items, with higher scores reflecting greater resilience (Connor & Davidson, 2003). Reliability of scores on the CD-RISC estimated using Cronbach’s *α* ranged from .89 to .94 (Hourani et al., 2012; Maguen et al., 2008; Pietrzak et al., 2010).

#### Perceived Stress

Each item in the PSQ is answered on a 4-point scale, ranging from 1 = *almost never* to 4 = *usually* (Levenstein et al., 1993). Scores are calculated by reverse scoring eight items and then summing all item scores, resulting in a raw score range of 30 to 120 (Levenstein et al., 1993). Because 30 is the minimum score, 30 points are subtracted from raw scores to create a rescaled score that is divided by 90 to standardize scores to a 0–1 range (0 = *no stress* to 1 = *highest stress*; Levenstein et al., 1993). The reported Cronbach’s *α* for scores on the PSQ were .80 to .93 (Fliege et al., 2005; Kocalevent et al., 2007; Levenstein et al., 1993).

### Data Analysis

IBM SPSS Statistics 23 was used for the statistical analysis. Initial analysis included descriptive statistics to characterize demographic status, military grade, past access to MH services, past deployment(s), and questionnaire scores. Because of the smaller number of minority participant responses, comparisons made by race were based on identification as White or non-White. Frequencies and proportions for stigma or barrier items were compared based on a stigmatizing or barrier response (*agree, strongly agree*) versus a nonstigmatizing or no barrier response (*strongly disagree, disagree, neither agree nor disagree*; Hoge et al., 2004). The magnitude and direction of associations among stigma and barriers to accessing MH services, stress, and resilience were assessed by Pearson correlation coefficients. Differences in stigma, barriers, stress, and resilience based on race, ethnicity, gender, military grade, and past deployment were examined using multivariate analysis of variance (MANOVA) with post hoc tests.

## RESULTS

### Sample Characteristics

Demographic characteristics for the sample are presented in Table 1. Approximately 1,397 USAF nursing personnel (505 RNs and 892 medical technicians) were assigned to the study sites. Two hundred fifty RNs (*n* = 141), medical technicians (*n* = 104), and respondents who did not disclose their position (*n* = 5) completed the survey. The response rate was 18%.

**TABLE 1:**
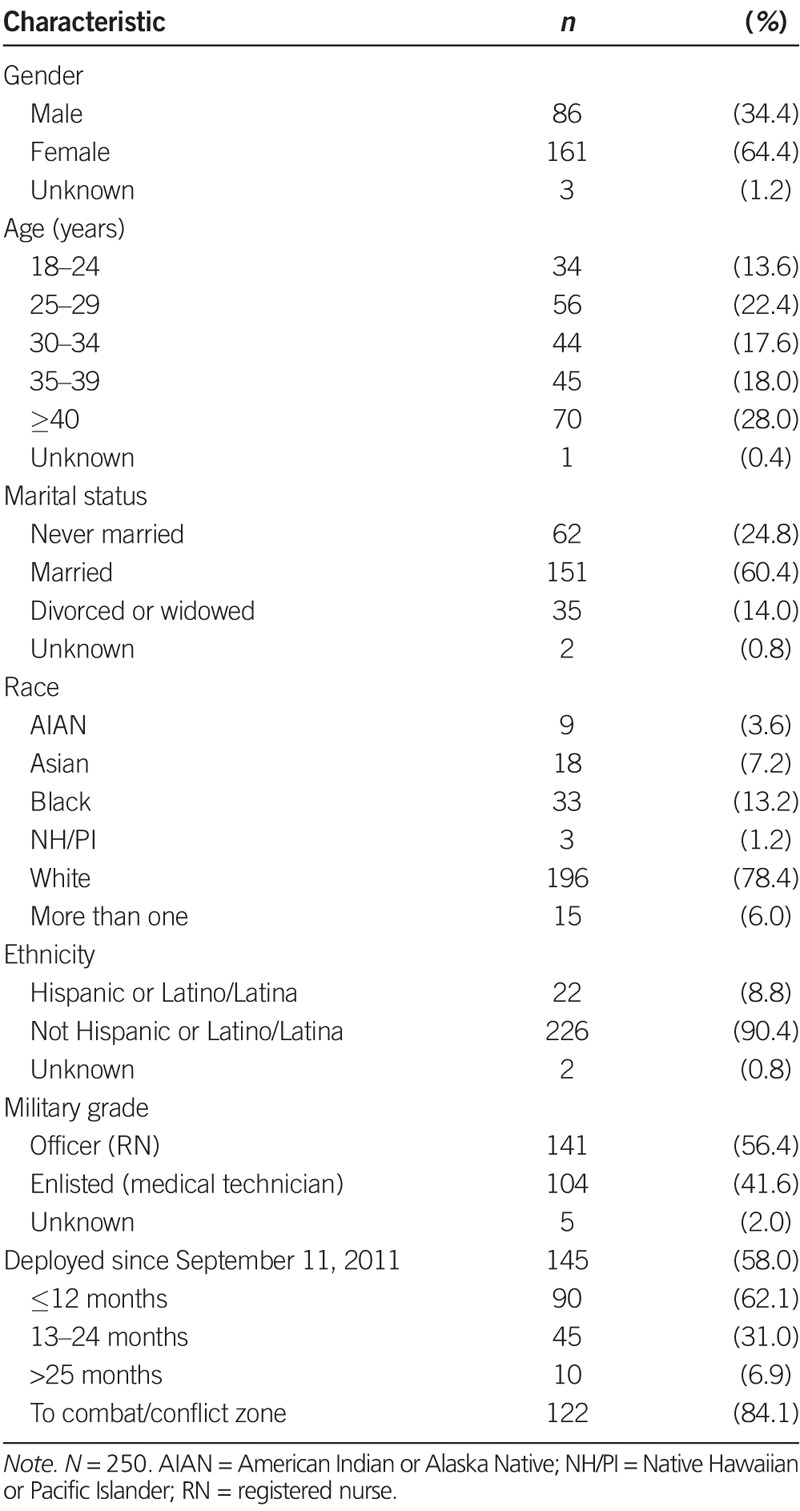
Participant Characteristics

### Levels of Stigma, Barriers to Care, Resilience, and Stress

Table 2 provides descriptive statistics for the Stigma and Barriers to Care scales, CD-RISC, and PSQ. Scores on all scales showed adequate reliability as estimated using Cronbach’s *α*. There were no significant differences in scores by study site for any of the scales. Mean scores were consistent with a response of *neither agree or disagree* for the Stigma Scale and *disagree* for the Barriers Scale. Individual items for the Stigma and Barriers scales were examined as both continuous and dichotomized scores (Table 3). Mean CD-RISC scores were consistent with an item level of *often*. The PSQ mean score was consistent with an item level between *sometimes* and *often*.

**TABLE 2:**
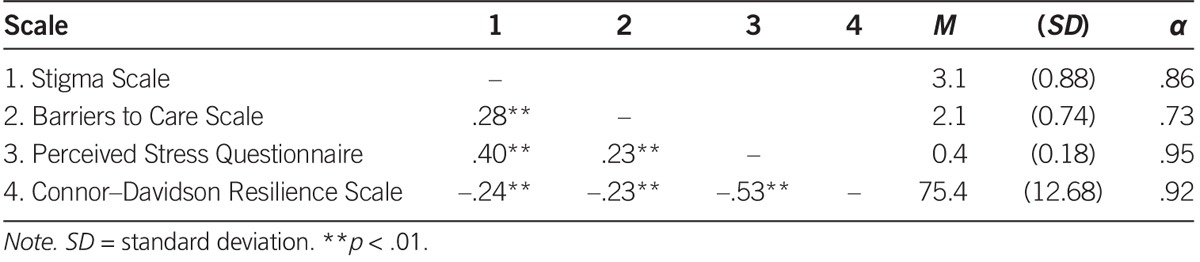
Stigma, Barriers to Care, Resilience, and Stress Scales: Descriptive Statistics and Correlations

**TABLE 3:**
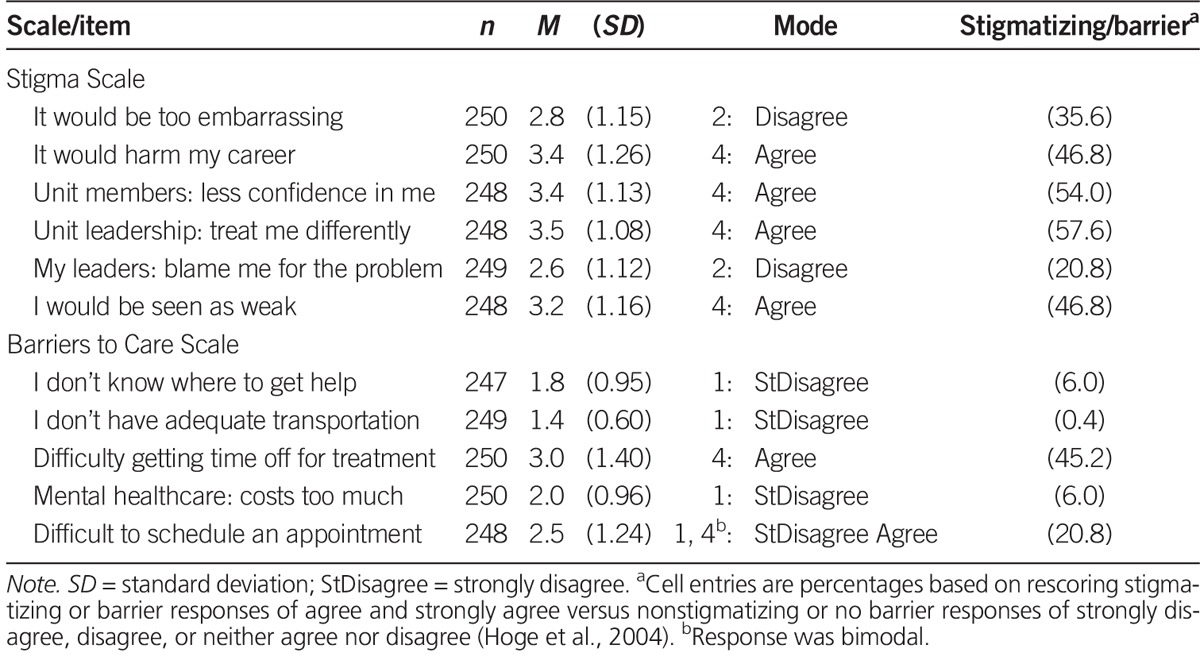
Stigma Scale and Barriers to Care Scale: Descriptive Statistics

### Associations Among Stigma, Barriers to Care, Resilience, and Stress

Significant relationships were found among stigma, barriers to care, resilience, and stress (Table 2). Resilience was weakly and negatively associated with stigma and with barriers to care. Barriers to Care scores were correlated positively but weakly with stress and moderately with stigma. A stronger, positive association was found between stress and stigma, and a strong, negative correlation was found between perceived stress and resilience.

### Demographic Characteristics and Stigma, Barriers to Care, Resilience, and Stress

The MANOVA analyses showed no significant interaction among gender, ethnicity, or race and no significant multivariate or univariate main effects of any of those variables on stigma, barriers, resilience, and stress—either jointly or separately. There was no significant difference between RNs and medical technicians in terms of previous deployment, and no significant multivariate interaction effect between rank and previous deployment status with respect to stigma, barriers, resilience, or stress. There was no significant multivariate effect or univariate main effect of deployment status on stigma, barriers, resilience, and stress—either jointly or separately.

Military grade accounted for approximately 10% of the variance overall in the composite of stigma, barriers, resilience, and stress (Wilks’ λ = .899, *F*[4, 230] = 6.47, *p* < .001). There were significant univariate main effects of RN versus medical technician status on stigma (*p* = .02), resilience (*p* = .004), and stress (*p* = .04), accounting for approximately 2% of the variance in stigma and stress and approximately 4% of variance in resilience. Two-sample *t*-tests were used as post hoc tests to generate Cohen’s *d* effect size estimates and 95% confidence intervals (CIs) for differences between RNs and medical technicians for each scale (Table 4). RNs reported significantly higher levels of stigma and resilience and significantly lower levels of stress compared with medical technicians. However, the effect sizes for these differences were small.

**TABLE 4:**
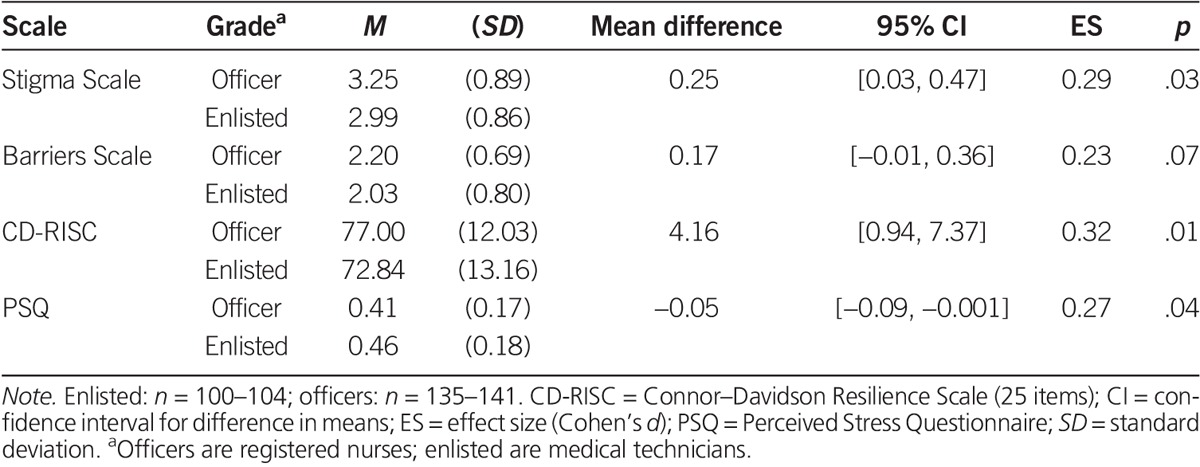
Stigma, Barriers, Resilience, and Stress by Military Grade

Because of differences in stigma scores based on military grade, a comparison was made between RNs’ and medical technicians’ dichotomized stigma items. Fisher’s exact tests were used to assess for differences in RNs’ versus medical technicians’ responses to each dichotomized stigma item. RNs were more likely than medical technicians to agree that accessing MH services *would be too embarrassing* (41.8% vs. 27.9%, respectively; difference = 13.9%, 95% CI [0.02, 0.25], *ϕ* = .14, *p* = .03) and that *unit leaders might treat me differently* (63.6% vs. 50.5%, respectively; difference = 13.1%, 95% CI [0.005, 0.25], *ϕ* = .13, *p* = .05).

## DISCUSSION

As in the study by Hernandez et al. (2014), substantial percentages of RNs and medical technicians agreed that seeking care for MH issues would be stigmatizing in terms of what unit peers and leaders might think of them and potential adverse consequences for career advancement. The proportions agreeing with those items were within ranges previously reported by combat veterans who screened positive for an MH concern (Gorman et al., 2011; Hoge et al., 2004; Kim et al., 2011). Concerns about stigma were more prevalent among RNs, despite higher levels of resilience and lower levels of stress compared with medical technicians. Concerns about barriers to seeking MH care were substantially less than concerns about stigma.

These findings are of concern for several reasons. First, USAF nursing personnel care for patients who experience stress or have MH issues. If caregivers themselves have concerns about the consequences of seeking MH care, they may communicate ambivalence to those they should be encouraging to get help. Second, nursing personnel receive formal education regarding the care of individuals experiencing an MH disorder. Therefore, nursing personnel’s concerns cannot easily be ascribed to ignorance about how military healthcare operates. In addition, concerns about stigma persist among USAF nursing personnel despite Department of Defense efforts to encourage service members to seek help for stress or psychological problems.

Consistent with expectations, stigma and barriers are positively correlated, although the relationship was weaker than previously reported among military service members who were not healthcare providers (*r* ≈ .4; Britt et al., 2011). As might be expected, there was a moderately strong inverse relationship between perceived stress and resilience, whereas resilience was inversely—although only weakly—correlated with stigma. In part, this may reflect the generally high levels of resilience reported by participants. The level of resilience was comparable to what has been reported among veterans who did not have PTSD or suicidal ideation (Green et al., 2010; Pietrzak et al., 2010, 2011; Pietrzak & Southwick, 2011). We also found that stigma was more strongly correlated with stress than with resilience. Although differences were not large, we found lower levels of resilience and higher levels of stress among medical technicians compared with RNs. It is conceivable that interventions focused on stress reduction and enhancing resilience may be of greater utility for enlisted nursing personnel.

### Limitations

This study had several limitations. First, because the survey was anonymous, we were unable to target follow-up reminders to nonrespondents. Consequently, our response rate was low, and the sample size was about 8% below the target. However, the sample size was adequate to detect statistically significant differences and correlations corresponding to small standardized effect sizes. Second, self-selection bias may have influenced responses. The REDCap system has the capacity to determine whether an individual had completed a survey; however, that functionality depends on the researcher having the e-mail distribution lists. Because of USAF policy and the need to protect anonymity, we were not able to house the e-mail lists. Therefore, we used an open link that was accessible to anyone who received the invitation to participate, and we cannot rule out the possibility that some individuals may have completed the survey more than once.

### Conclusion

This was the first known multisite study to assess stigma and barriers to accessing MH services, stress, and resilience among USAF RNs and medical technicians. Future studies should investigate stigma, barriers, resilience, and stress among nursing personnel in other service branches and other categories of military healthcare providers. A broader base of research in these areas will be vital for informing the development of evidence-based interventions to reduce stigma to seeking MH services among military healthcare providers and support “care for the caregiver.”

## Figures and Tables

**Figure:**
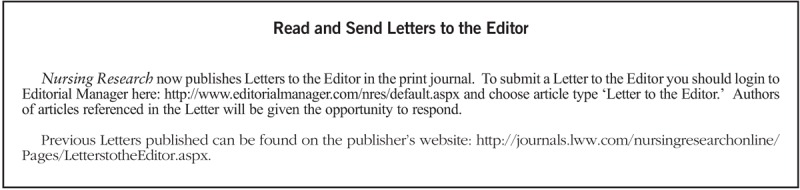
No caption available.
